# Fetal and neonatal outcome in severe alloimmunization managed with intrauterine transfusion: 18-year experience in a tertiary referral hospital in China

**DOI:** 10.3389/fped.2023.1157004

**Published:** 2023-04-12

**Authors:** Wenxu Pan, Haiyan Wu, Junlin Chen, Xinyue Mo, Hongxin Wang, Qun Fang, Yijuan Li, Yuefang Huang

**Affiliations:** ^1^ Department of Pediatrics, First Affiliated Hospital, Sun Yat-sen University, Guangzhou, China; ^2^ Fetal Medicine Centre, Department of Obstetrics, First Affiliated Hospital, Sun Yat-sen University, Guangzhou, China

**Keywords:** alloimmunization, hemolytic disease of the fetus and newborn, intrauterine transfusion, hydrops, anemia, outcomes

## Abstract

**Background:**

Hemolytic disease of the fetus and newborn (HDFN) due to red cell alloimmunization, is an important cause of fetal and neonatal morbidity and mortality. However, fetal and neonatal outcome of HDFN managed with intrauterine transfusion (IUT) in China are unknown. In addition, fetal and neonatal outcomes according to the type of maternal red cell alloantibodies involved and outcomes of hydrops fetalis are also unclear.

**Objectives:**

The objective of this study was to evaluate fetal and neonatal outcomes of severe red-cell alloimmunization treated by IUT, to compare the outcomes according to the type of antibody, and to investigate the perinatal and postnatal outcomes of hydrops fetalis due to red cell alloimmunization.

**Methods:**

A retrospective study of pregnancies affected by HDFN and managed with IUT at a tertiary care university hospital in China between January 2001 and December 2018 was performed. Fetal and neonatal outcomes were investigated, and comparison of outcomes depending on the type of antibody and comparison of outcome between hydrops fetalis and fetuses without hydrops were also conducted.

**Results:**

244 IUTs were performed in 81 fetuses from 80 pregnancies. Anti-RhD was the major etiology of HDFN requiring IUT (71.6%). The fetal survival rate was 90.1%. The survival rate of the hydropic fetuses was significantly lower than those of the non hydropic fetuses (61.2% vs. 95.6%) (*P* = 0.002**). Compared with non hydropic fetuses, hydropic fetuses had significantly lower gestational age and lower hemoglobin level at first IUT. The neonatal survival rate was 98.6%. Exchange transfusions were required in 26% of the neonates. 30.1% of neonates had late anemia and required top-up transfusions, and hydropic fetuses required more late top-up transfusions than fetuses without hydrops. No significant difference in fetal and neonatal outcomes was found among the four subgroups stratified by the antibody involved.

**Conclusion:**

Our study demonstrates that IUT is an effective and safe therapy for severe HDFN at our institution. Early detection and treatment of hydrops is critical for perinatal outcomes. Particular attention should be paid to late postnatal anemia in affected neonates and top-up transfusion is still commonly needed.

## Background

Hemolytic disease of the fetus and newborn (HDFN) results from an incompatibility between maternal and fetal erythrocyte antigens. This occurs when fetal and neonatal erythroid cells are destroyed by maternal erythrocyte alloantibodies that cross the placenta to the fetal circulation (1). If untreated, the fetal and neonatal prognosis of HDFN is poor, it can lead to fetal anemia, hydrops and even intrauterine death, and postnatally to hyperbilirubinemia and kernicterus of the newborn.

HDFN is most commonly caused by anti-D alloantibody. The routine administration of prophylactic anti-D immunoglobulin to D- women during pregnancy and shortly after the birth of D + neonates has dramatically reduced the incidence of HDFN due to anti-D. However, the availability of anti-D immunoglobulin in developing countries is limited, RhD hemolytic disease remains a significant cause of fetal and neonatal morbidity and mortality. In addition, routine antenatal screening for irregular antibodies was not feasible and necessary in China due to very low prevalence of HDFN caused by non-RhD alloimmunization. However, non-RhD alloantibodies including anti-E, anti-c, and anti-M have been reported to cause severe HDFN in Chinese population (2–4).

Intrauterine transfusion (IUT) has become the cornerstone of treatment for fetal anemia due to red-cell alloimmunization and has significantly improved perinatal outcomes in the past decades (5). Nowadays, IUT is considered a safe procedure in experienced hands. With the use of this life-saving treatment, the survival rate of fetuses with and without hydrops fetalis has increased to 75% and almost 90%, respectively (6, 7).

Previous studies regarding perinatal outcomes of severe red-cell alloimmunization treated by IUT have mainly focused on perinatal survival rates after IUT, and most of these studies were carried out in developed countries (7–9). Few studies have investigated the effect of IUT on the clinical outcome of the neonate after birth (8, 10). Moreover, very limited information was available regarding the fetal and neonatal outcomes of HDFN managed with IUT in developing countries, and there have been no published reports of the impact of IUT on fetal and neonatal outcomes of HDFN in China. In addition, fetal and neonatal outcomes according to the type of alloantibody involved and outcomes of hydrops fetalis associated with red cell alloimmunization are also unclear. Thus, the aim of this study was to evaluate fetal and neonatal outcomes of severe red cell alloimmunization treated by IUT, to further investigate the outcomes depending on the type of alloantibody and outcomes of hydrops fetalis due to red cell alloimmunization at a tertiary care university hospital in China.

## Methods

A retrospective study was carried out in the NICU of First Affiliated Hospital, Sun Yat-sen University in Guangzhou, a developed region located in the south of China. The hospital is one of the largest tertiary university hospitals in the country, and the fetal medicine unit at this hospital is a tertiary referral unit that manages pregnancies affected by HDFN throughout China.

Pregnant women were screened for alloantibodies based on the following criteria: (1) having Rh(D) negative phenotype; (2) having previous adverse pregnancy outcomes, including recurrent abortion, fetal demise, and hydrops fetalis; (3) identification of unexpected alloantibodies in ABO blood typing; and (4) having a previous history of blood transfusion (11). Pregnancies affected by a raised antibody titre are monitored by serial Doppler measurements to assess the middle cerebral artery peak systolic velocity (MCA-PSV). Severe fetal anemia was defined as MCA-PSV ≥ 1.5 MoM, and then fetal blood sampling is performed, and the treatment with IUT is carried out. The IUT procedure has been described previously (12). IUT was performed until the 34th week of gestation.

Perinatal and neonatal data of patients who underwent IUT treatment for fetal anemia due to red cell alloimmunization at our hospital between January 2001 and December 2018 were retrospectively collected and reviewed. During the study period, a total of 255 IUTs were performed in 85 fetuses with severe anemia due to red blood cell alloimmunization. Because we aim to investigate minor blood group incompatible hemolytic diseases treated with IUT, four patients who underwent 11 IUTs for HDFN due to ABO incompatibility were excluded from the study. Only 81 fetuses who underwent IUT because of RhD or non-RhD alloimmunization (excluding ABO incompatibility) were included in this study. The study was approved by the Ethics Committee of First Affiliated Hospital, Sun Yat-sen University.

Perinatal and neonatal data were collected from the patient's medical notes at this hospital. The perinatal data obtained included maternal age of first IUT, parity, antibody titres and gestational age (GA) of the fetus at first IUT, the presence of hydrops fetalis, the results of fetal blood sample (antibody titer, fetal haemoglobin and bilirubin level), number of IUT received during pregnancy, mode of delivery. Hydrops was defined as the accumulation of excess fluid in the fetus, producing the following signs: generalized edema, ascites, and pleural or pericardial effusions (13).

The neonatal data collected included gestational age and weight at birth, APGAR score, cord blood results (antibody titer, haemoglobin and bilirubin level and umbilical cord blood gas analysis), serial monitoring of infant haemoglobin and bilirubin level, type of alloimmunization, exchange transfusion requirement, top-up transfusion requirement, duration of phototherapy, non-hematological neonatal complications, and total length of hospital stay. Early top-up transfusion was defined as blood transfusion before three completed postnatal weeks, and late top-up as transfusion needed at or after three completed weeks (10). The indications for phototherapy, exchange transfusion, and top-up transfusion treatments in neonates with HDFN managed with IUT were as previously described (14).

Following data collection, Procedure-related complications were also reviewed. Procedure-related complications were classified as follows: premature rupture of membranes; preterm delivery; intrauterine infection within the first seven days after IUT; emergency caesarean section because of fetal distress within the first 24 h after IUT; fetal loss and neonatal death after IUT (15).

Outcome measures included fetal and neonatal survival treated by IUT, the need for phototherapy, exchange transfusion and top-up transfusion after IUT in this study cohort. In addition, we compared the fetal and neonatal outcome of maternal RBC alloimmunizations according to the type of antibody (isolated anti-D, anti-D + other Rh, other Rh and MN alloimmunization), and also compare the outcome of hydrops fetalis with fetuses without hydrops.

### Statistical analysis

Statistical analysis was performed using Statistical Package for the Social Sciences Version 25.0 (SPSS, Chicago, IL, USA). Unpaired t-test was used to analyze gestational age at 1st IUT, hemoglobin before 1st IUT, gestational age at birth, birth weight, hemoglobin at birth, TBIL at birth. Mann-Whitney U test was used to analyze number of IUTs, phototherapy duration, number of exchange transfusions, number of top-up transfusions. Categorical variables like fetal survival rate and the rate of 1st IUT ≤ 20 weeks were analyzed with *χ*^2^ statistics or Fisher's exact test. The qualitative data were analyzed by fisher's exact test and quantitative data were analyzed by Kruskal-Wallis test when we compare the fetal and neonatal outcomes among four groups with different antibody types. A Spearman correlation coefficient was used to evaluate the relation between the number of IUTs and the phototherapy duration, number of exchange transfusions and RBC transfusions. A *P* value <0.05 was considered statistically significant.

## Results

### Characteristics of fetuses managed with IUT

During the study period, 244 IUTs were performed in 81 fetuses from 80 pregnancies with red blood cell alloimmunization. The sensitizing antigen was D in 71.6% (58/81) of cases, a combination of antigen D and C in 14.8% (12/81), antigen D and E in 6.2% (5/81), antigen M in 3.5% (3/81), antigen E in 2.5%(2/81), and a combination of antigen E, c and Jkb in 1/81 (1.2%).

The mean maternal age at first transfusion was 30.5 (±4.2) years. The median (range) peak maternal antibody titers were 1:768 (4 to 16384). The median (range) number of IUTs was 3(1 to 8). The mean GA at first IUT was 26.9 (±4.9) weeks. The mean hemoglobin (Hb) at first IUT was 69.8(±24.2) g/L. Thirteen of the 81 fetuses were diagnosed with hydrops fetalis. There were 8 fetal losses, therefore the fetal survival rate was 90.1% (73/81).

### Characteristics and interventions of neonates treated with IUT

Of the 73 neonates, 57 were preterm neonates (<37 gestational weeks), while the remaining 16 were full-term neonates (≥37 gestational weeks). only three (4%) were born at less than 32 weeks. The mean GA at birth was 35.5 (±1.8) weeks, The mean BW was 2,648 (±466) g. The most common mode of delivery was elective cesarean section (82.2%, 60 out of 73). The mean serum total bilirubin (TBIL) level at birth was 108.8 (±35.6) µmol/L. The mean maximal serum TBIL level was 206.4 (±73.7) µmol/L. The mean hemoglobin (Hb) at birth was 112.6 (±21.6) g/L. All infants were treated with phototherapy, the median (range) duration of phototherapy was 112 h (63 to 312). Exchange transfusions were required in 19 (26%) of the 73 infants. Top-up transfusions were required in 65 (89%) of these infants. Early transfusion was required in 64 infants and late in 22 infants.

One preterm infant of 35 weeks gestation died on day 15 of life due to severe hypoxic ischemic encephalopathy and coagulopathy, therefore the neonatal survival rate was 98.6% (72/73).

### Comparison of fetal and neonatal outcomes depending on the type of antibody

Data on the comparison of fetal and neonatal outcomes depending on the type of antibody are given in Table 1. Fetal outcomes included hydrops fetalis, first IUT before 20 weeks of gestation, Hb before first IUT, number of IUTs and fetal survival rate. No significant difference in fetal outcome was found among the four subgroups of patients with HDFN (anti-D, anti-D with non-anti-D Rh, non-anti-D Rh, anti-M) stratified by the antibody involved. Regarding neonatal outcomes, there was no difference among the four subgroups in the mean GA at birth, mean BW, Apgar score <7 at 1 min and 5 min, mean Hb at birth, mean serum TBIL level at birth, mean maximal serum TBIL level, and the duration of phototherapy, the need for exchange transfusions and top-up transfusions. Interestingly, we noted that no patients in non-anti-D group and anti-M group required exchange transfusion, but the sample size in each subgroup was small.

**Table 1:** Comparison of outcomes depending on the type of antibody.

	Anti-D Rh (*n* = 58)	Anti-D + non-Anti-D Rh (*n* = 17)	Non-Anti-D Rh (*n* = 3)	MN (*n* = 3)	*P* value
**Fetal outcomes**					
Hydrops	10 (17.2%)	3 (17.6%)	0 (0%)	0 (0%)	0.705
1st IUT ≤ 20 weeks	6 (10.3%)	3 (17.6%)	1 (33.3%)	0 (0%)	0.370
Hemoglobin at 1st IUT, g/L	70.1 ± 23.5	67.2 ± 25.3	70.7 ± 13.5	57.0 ± 31.1	0.073
No. of IUTs	2 (1,7)	4 (1,8)	4 (3,7)	5 (2,8)	0.776
Fetal survivors	51 (87.9%)	16 (94.1%)	3 (100%)	3 (100%)	0.829
**Neonatal outcomes**					
Gestational age at birth, week	35.5 ± 1.9	35.2 ± 2.1	34.0 ± 1.5	36.0 ± 0.1	0.592
Birth weight, g	2637 ± 475	2537 ± 517	2393 ± 514	2963 ± 348	0.428
Apgar < 7, 1 min	4 (7.8%)	3 (18.8%)	2 (66.7%)	0 (0%)	0.053
Apgar < 7, 5 min	0 (0%)	0 (0%)	0 (0%)	0 (0%)	NA
Hemoglobin at birth, g/L	111.5 ± 20.3	115.2 ± 28.5	121.7 ± 14.3	106.7 ± 4.5	0.692
TBIL at birth, umol/L	114.1 ± 38.9	103.3 ± 22.2	91.6 ± 37.5	75.5 ± 3.5	0.155
Maximal TBIL, umol/L	210.7 ± 72.1	207.2 ± 84.2	140.1 ± 64.8	204.2 ± 52.4	0.439
Phototherapy duration, h	120 (63,312)	96 (69,248)	141.5 (87,196)	104 (96,112)	0.447
Exchange transfusion	14 (27.5%)	5 (31.3%)	0 (0%)	0 (0%)	0.705
No. of top-up transfusions	2 (0,6)	2.5(1,5)	3(1,5)	2(2,4)	0.455

Data are expressed as mean ± SD, median (range) or number of infants (percentage).

IUT, intrauterine transfusion; TBIL, total bilirubin.

### Comparison of fetal and neonatal outcomes between hydropic fetuses and fetuses without hydrops

Data on the comparison of fetal and neonatal outcomes between hydropic fetuses and fetuses without hydrops was shown in Table 2. The most striking difference was observed with respect to fetal outcomes: compared with non hydropic fetuses, the hydropic fetuses had significantly lower gestational age and lower Hb level before first IUT, higher rate of IUT performed before 20 weeks of gestation and significantly lower fetal survival rate. Regarding neonatal characteristics at birth, there was no difference between these two groups in the mean GA at birth, mean BW, mean Hb at birth and mean serum TBIL level at birth. With respect to neonatal management, hydropic fetuses required more late top-up transfusions than fetuses without hydrops, but no significance was found between these two groups in the duration of phototherapy and the need for exchange transfusions.

**Table 2:** Comparison of outcomes between hydrops and non-hydrops.

	Hydrops (*n* = 13)	Non-hydrops (*n* = 68)	*P* value
**Fetal characteristics**			
Gestational age at 1st IUT, week	23.5 ± 3.6	27.7 ± 4.9	0.002**
1st IUT ≤ 20 weeks	4 (30.8%)	6 (8.8%)	0.049*
Hemoglobin before 1st IUT, g/L	52.9 ± 24.9	73.1 ± 22.8	0.014*
No. of IUTs	3 (1,7)	3 (1,8)	0.369
Fetal survival rate	8 (61.2%)	65 (95.6%)	0.002**
**Characteristics at birth**			
Gestational age at birth, week	35.8 ± 1.7	35.4 ± 1.8	0.496
Birth weight, g	2748 ± 556	2635 ± 457	0.521
Hemoglobin at birth, g/L	99.8 ± 29.2	113.8 ± 20.6	0.298
TBIL at birth, umol/L	106.2 ± 26.5	109.1 ± 36.6	0.821
**Neonatal management**			
Phototherapy duration, h	123.5 (69,144)	112 (9,312)	0.860
No. of exchange transfusions	3 (23.1%)	16 (23.5%)	0.421
No. of top-up transfusions	2.5 (0,6)	2 (0,4)	0.14
Early top-up transfusions	1.5 (0,4)	1 (0,4)	0.654
Late top-up transfusions	1.5 (0,2)	0(0,2)	0.012*

Data are expressed as mean ± SD, median (range) or number of infants (percentage).

IUT, intrauterine transfusion; TBIL, total bilirubin.

*P < 0.05 and **P < 0.01 vs. non-hydrops.

### Correlation between IUTs and neonatal management

A negative correlation was noted between the number of IUTs and the duration of phototherapy (*r* = −0.448, *P* < 0.01) while a positive correlation was found between the number of IUTs and the number of late top-up transfusions (*r* = 0.266, *P* < 0.05), but not with the number of exchange transfusions.

### Procedure-related complications

The total procedure-related complications rate was 4.5% (11/244 procedures), and the fetal loss rate per procedure was 1.6% (4/244 procedures). Three cases of emergency caesarean section, 4 cases of preterm delivery, and 4 cases of fetal loss after IUT were classified as procedure-related complications.

## Discussion

To the best of our knowledge, this is the first retrospective study on the fetal and neonatal outcome in red blood cell alloimmunization treated with IUT in China. Although it is a single-center study, our fetal medicine center is one of the oldest and largest centers of IUT treatment for HDFN in China. Therefore, the majority of the pregnant women with red blood cell alloimmunization referred to our center are from different provinces throughout China. The fetal survival rate after transfusion in our study was 90.1%, which is comparable to that reported by developed countries (7, 14, 16). The fetal survival rate was slightly higher than recent reports from developing countries (77.5%–82%) (17, 18). In this study, the earliest gestational age of the fetus at 1st IUT was 16 weeks, and the lowest Hb before IUTs was 18 g/L. These two fetuses survived with satisfactory outcomes. Such success is attributable to the effective IUT treatment for the severely affected fetuses. Our findings showed that the rate of procedure-related complications was 4.5%, and the fetal mortality rate per procedure was 1.6%. This latter rate is below the 2%, as suggested in the NICE guidance (19).

Our study showed that anti-D accounted for a great proportion of red blood cell alloimmunization (71.6%) managed with IUT. In many developed countries, management guidelines have been developed to prevent D immunization (19), leading to a substantial decrease of D immunization (16% to 0.17%–0.28%) (20), associated with a significant reduction in mortality in relation to HDFN. However, Rh-D alloimmunization remains the most common indication for IUT therapy in our country because of the lack of routine antenatal and postpartum use of anti-D Ig as immunoprophylaxis. Compared with Rh-D alloimmunization, non-D Rh alloimmunization is less well known and studied less. In this study, we found that two cases of anti-RhE and three cases of anti-M were associated with the need for IUT. It is reported that anti-E is associated with mild to moderate HDFN (21). However, some authors have reported cases of significant HDFN or of hydrops fetalis and the need for IUT due to anti-E alloimmunization (22). The incidence of HDFN due to anti-M was reported to be low and reports of clinically significant HDFN are rare. In two large case series of the anti-M positive pregnancies, none had fetal anemia (23, 24). However, some recent studies have published cases of hydrops fetalis, stillbirth, neonatal death and severe neonatal consequences associated with anti-M alloimmunization (25, 26). In this study, two infants with maternal anti-M antibody titers of 8 or 4 at the beginning of IUT intervention experienced severe HDFN, suggesting anti-M antibody may result in severe HDFN at a lower maternal antibody titer. In addition, whether the different types of maternal antibodies responsible for fetal anemia managed with IUT may influence treatment and outcomes is unclear, furthermore, whether anti-D alloimmunizations combined with other Rh associated antibody may have more adverse outcomes is also unknown. Very limited study has compared the management and fetal and neonatal outcome of maternal alloimmunization requiring IUT according to the type of antibody (27). Thus, we also performed a comparative study on management and perinatal outcome of maternal RBC alloimmunization requiring IUT depending on the type of antibody. The results of our study showed that there were no differences in the rate of hydrops fetalis or the rate of fetal survival, the duration of phototherapy, and the need for exchange transfusion and top-up transfusions among four groups (Isolated anti-D, anti-D + other Rh, other Rh and MN). With respect to these outcomes, our findings were similar to those of Phung et al. (27). However, in the study by Phung et al., the patients were classified according to the different antibody types including anti-D, other Rh (anti-c and anti-E), and anti-K1. We also noted that there was no hydrops fetalis, and no death case in other Rh group or MN group. Due to small sample size in other Rh group and MN group, additional data are needed to compare perinatal outcome of maternal RBC alloimmunization requiring IUT according to the antibody involved.

Recent data about red blood cell alloantibodies in patients from mainland China showed that anti-D was the most common antibody (82/122, 67.2%) (11). This finding, together with our results suggests that antenatal and postpartum Rh prophylaxis should become a routine approach in our country. Moreover, HDFN caused by antibodies other than anti-D should also be given sufficient attention. Close prenatal observation, prompt diagnosis and management are especially important for suspected HDFN with a previous unexplained fetal demise or spontaneous abortion. Intrauterine transfusion therapy is required if fetal anemia is detected by measurements of MCA-PSV.

Hydrops complicated by severe hemolytic disease is a well-known risk factor for unfavorable fetal outcomes. However, there are limited reports investigating fetal survival after IUT for fetal hydrops due to alloimmunization. In the present study, the survival rate after IUT for hydropic and non hydropic fetuses was 61.2% and 95.6%, respectively, which is comparable to previous studies (6, 28). Our study also provided evidence that the survival rate was significantly lower in the hydropic fetuses than in the non hydropic fetuses. Apart from the fetal survival rate, data are lacking on the comparison of other fetal and neonatal outcomes of hydropic fetuses with non hydropic fetuses treated with IUT. The results of our study showed that compared with non hydropic fetuses, hydropic fetuses had significantly lower gestational age, lower hemoglobin level at first IUT and higher proportion of patients requiring first IUT before 20 weeks of gestation. Regarding postnatal management, more of the hydropic fetuses required late top-up transfusions. Therefore, early referral of patients to experienced perinatology centers and timely detection and treatment are essential to prevent hydrops and improve outcome of early severe red cell alloimmunization.

The effect of IUT on postnatal phototherapy, exchange transfusions and top-up transfusions remains uncertain. Janssens et al. found that a greater number of IUTs significantly decreased the need for phototherapy (29). But the study by Laura et al. did not find any relevance between the number of IUTs, postnatal phototherapy, exchange transfusion and top-up transfusion (30). In the present study, we found a significant negative correlation between the number of IUTs and the duration of phototherapy, which was similar to the study by Janssens et al. (29). Moreover, a significant relationship was noted between the number of IUTs and subsequent need for top-up transfusion to treat late anemia in this study. The inconsistent results may be related to lack of consensus around indications for exchange transfusions, top-up transfusion and optimal use of phototherapy following IUT therapy.

The majority of neonates with HDFN managed with IUT develops late-onset anemia between the second and the sixth week of life. In this study, late anemia requiring at least one top-up transfusion occurred in 30.1% of the infants. The pathophysiologic mechanism associated with late hyporegenerative anemia is still unclear. Ongoing intramedullar hemolysis (31), marrow suppression from IUT (32), erythropoietin (EPO) deficiency (33), and direct inhibitory effect of anti-D on erythroid progenitors (34) have been discussed as causative mechanisms of late hyporegenerative anemia.

Our study limitations include being retrospective and small sample size in non-RhD alloimmunization. Additional prospective studies are needed to explore and compare perinatal outcomes of maternal RBC alloimmunization treated with IUT according to the antibody involved.

In conclusion, our study demonstrates that IUT is an effective and safe therapy for severe fetal anemia resulting from red blood cell alloimmunization. The overall survival is comparable with previous studies from developed countries. Anti-D remains the most common antibody in fetuses requiring IUT, suggesting the importance of routine antenatal and postpartum use of anti-D Ig as immunoprophylaxis. Early detection and referral of hydrops is critical for perinatal outcomes. Particular attention should be paid to late anemia in affected neonates, and top-up transfusion is still commonly needed. Further studies are needed to compare perinatal outcomes of maternal RBC alloimmunization requiring IUT according to the antibody involved.

## Data Availability

The raw data supporting the conclusions of this article will be made available by the authors, without undue reservation.
